# Influence of Tooth-Brushing on Early Healing after Access Flap Surgery: A Randomized Controlled Preliminary Study

**DOI:** 10.3390/ma14112933

**Published:** 2021-05-29

**Authors:** Carlo Bertoldi, Luigi Generali, Pierpaolo Cortellini, Michele Lalla, Sofia Luppi, Aldo Tomasi, Davide Zaffe, Roberta Salvatori, Stefania Bergamini

**Affiliations:** 1 Department of Surgery, Medicine, Dentistry and Morphological Sciences with Transplant Surgery, Oncology and Regenerative Medicine Relevance, University of Modena and Reggio Emilia, 41124 Modena, Italy; aldo.tomasi@unimore.it (A.T.); stefania.bergamini@unimore.it (S.B.); 2 The European Research Group on Periodontology (ERGOPerio), 3855 Brienz-Bern, Switzerland; sandro@cortellini.org; 3 Department of Economics Marco Biagi, University of Modena and Reggio Emilia, 41121 Modena, Italy; michele.lalla@unimore.it; 4 Independent Researcher, 41124 Modena, Italy; sofialuppi@outlook.it; 5 Department of Biomedical, Metabolic and Neural Sciences, University of Modena and Reggio Emilia, 41125 Modena, Italy; 6 Biomaterials Laboratory, Department of Medical and Surgical Sciences of Children and Adults, University of Modena and Reggio Emilia, 41124 Modena, Italy; roberta.salvatori@unimore.it

**Keywords:** periodontal supportive therapy, chlorhexidine, oral ecosystem, chemical plaque control, mechanical plaque control, periodontal index

## Abstract

In the present study, the clinical outcomes obtained using three different protocols of post-operative plaque control for the 4 weeks after surgery were compared. Thirty healthy subjects, presenting at least one periodontal pocket requiring resective surgery, were selected and randomly distributed to three different groups corresponding to respective post-surgical protocols: (A) toothbrushes + chlorhexidine + anti-discoloration system (ADS + CHX); (B) toothbrushes + chlorhexidine (CHX); (C) only toothbrushes. The full-mouth plaque score (FMPS), full-mouth bleeding score (FMBS), probing pocket depth (PPD), recession depth (REC), clinical attachment level (CAL), and bleeding on probing (BoP) were measured in six aspects per tooth (mesio-buccal (MB), buccal (B), disto-buccal (DB), disto-lingual (DL), lingual (L), and mesio-lingual (ML)) at baseline, 3 months, and 6 months after surgery. FMPS and FMBS did not significantly change (*p* > 0.05), whereas PPD and CAL significantly decreased, and REC significantly increased in all groups during the study (*p* < 0.05). Clinical results were satisfactory in all cases, with no significant differences between groups 3 months after surgery. Six months after surgery, only PPD-MB was significantly different in the three groups (*p* < 0.05). Nevertheless, this value was not clinically relevant because the value of PPD-B (about 2 mm) in group C was physiologic. The mechanical plaque control was proven to be fundamental and sufficient in all the six aspects per tooth to guarantee an excellent clinical outcome without the need of chemical plaque control.

## 1. Introduction

Periodontal diseases are oral infections characterized by gingival inflammation, clinical attachment loss, and alveolar bone resorption [1,2,3].

Microbiological and histopathologic observations of diseased human tissues have been used to describe models of pathogenesis of periodontitis [4,5]. In addition, specific bacteria and immuno-inflammatory mechanisms have been implicated to build up new theoretical models of the pathogenesis of periodontitis, which include the microbiota activation of immuno-inflammatory pathways inducing the loss of periodontal attachment, leading to periodontal pocket formation [6].

In actuality, substantially little is known in inflammatory alterations and pathogenic mechanisms involved in periodontal disease onset, and therefore cause-related therapy, aiming to correct the causes that lead to periodontal disease, is a necessary process, with it focusing on controlling the “external” risk and prognostic factors, such as microbiota or lifestyles (e.g., smoking). 

The main aim of periodontal therapy is to stop the course of periodontal disease and to control the possible risk factors jointly interested in the course of this inflammatory disease. The cause-related therapy results effective when plaque control is maintained by patients and it is effectively maintainable in all sites affected by periodontal disease. 

Periodontal surgery is an adjunctive therapy that permits successful treatment of periodontal defects (e.g., deep pockets) that respond less favorably or would be unpredictable only through the cause-related therapy [7,8,9,10]. The surgical approach brings the periodontal condition back to a clinical condition that is able to be controlled by the supportive periodontal therapy. This is a cause-related therapy that has to be performed over time for periodontal patients in clinical burnout, and the same goal of the cause-related therapy is followed after surgery in order to control the external risk and prognostic factors. Regenerative periodontal therapy has been mainly developed in the past two decades. A large number of specific techniques and materials are used for this purpose, and there is quite often no agreement on the most effective surgical approach with respect to periodontal defects suitable for regenerative therapy. Flap surgery with osseous reshaping is a well-known method used to obtain predictive periodontal pocket resolution with a limited number of methodological variables. Moreover, the use of biomaterials is not usually considered. 

The use of chlorhexidine (CHX) after surgery is routine in periodontology. Substantial clinical evidence indicates that the healing process after periodontal surgery is favored by mechanical and/or chemical microbiota (e.g., plaque) control. Plaque control limits the infections, which are fearful post-operative complications. Infection could be expressed as excessive swelling, deposition of fibrin, margin necrosis, and flap mobilization, jeopardizing flap healing. Usual clinical indications for home dental care to patients after flap surgery include the use of chlorhexidine mouth rinsing 1 to 4 weeks postoperatively, with mechanical plaque control provided by the toothbrush and interdental cleaning devices re-introduced at variable times after the surgery [11,12,13,14,15,16,17,18,19].

The main problem was that non-professional use of ordinary dental brushes or dental floss could easily damage the healing soft tissues, which not yet adequately stabilized and structured. However, the use of CHX is burdened by some side effects, mainly related to stains, alterations in taste and erythematous–desquamative lesions of the oral mucosa, parotid swelling, and increase of calculus formation in long-term use. Among them, the most frequent is brown pigmentations that appear on the dental surfaces, prosthetic and composite restorations, and tongue after prolonged use, whereas others are rare, if not exceptional [20,21,22,23,24]. The evidence that these approaches offer tangible benefits, however, remains debatable [25]. The tooth surface increasing roughness linked to pigmentation increases plaque deposition. Pigmentations quite often require an early, strong professional dental hygiene of the teeth involved in the surgical site, thus jeopardizing surgical treatment advantages. On the other hand, plaque control performed by mechanical procedure is largely carried out long since, and it is considered greatly significant because it also allows for the physical breakdown of the structured oral ecosystems [26]. The chemical plaque control could play an additional role or even no clinical role.

Considering the respective development of periodontal surgery, of periodontal supportive therapy of late years, and the side effects of CHX, also leading to jeopardization of clinical outcomes obtained, the post-surgical use of mouth rinses with CHX has to be contextualized. Currently, two clinical protocols are followed, largely based on periodontists’ experience, but not on clinical trials. Some post-surgical protocols suggest a week of chemical plaque control in the absence of mechanical plaque removal, followed by the re-introduction of toothbrush and interdental cleaning at variable periods. Other protocols suggest a combined mechanical and chemical plaque control regimen immediately after surgery [13,14,27].

At present, the evidence does not allow for an evaluation of the efficacy, predictability, and safety of the two different conceptual approaches. Moreover, the lack of studies that consider the exclusive use of mechanical plaque control, performed in relation to up-to-date periodontal surgical techniques and modern dental-hygiene techniques, is glaring.

The aim of this randomized controlled clinical trial was to define the role of the mechanical plaque control through comparing the clinical outcomes obtained after periodontal surgery using three different protocols of postoperative plaque control for 4 weeks after surgery: (A) post-surgical toothbrushes +0.12% chlorhexidine-based oral rinse with an anti-discoloration system (ADS + CHX); (B) post-surgical toothbrushes +0.12% chlorhexidine-based oral rinse (CHX); (C) only post-surgical toothbrush.

## 2. Materials and Methods

### 2.1. Study Population and Design

All procedures of this clinical study, performed at the Modena University Hospital (Periodontology Unit of Dentistry and Oral-Maxillofacial Surgery), were approved and supervised by the local ethical committee of the Health Service of the Emilia-Romagna region (University Hospital of Modena, protocol number 4406/CE, registration number 369/17). An informed consent form, detailing all procedures of the study, as requested by the Helsinki protocols [28], was signed by all enrolled subjects.

Selected subjects were enrolled between December 2017 and October 2018. Healthy patients affected by moderate to advanced periodontitis were screened for the presence of at least one periodontal pocket associated with an intrabony defect. At the screening, the inclusion criteria were as follows: adult patients (underage and patients over age 70 were excluded), non-pregnant or lactating, non-smokers, and those without a history of alcohol abuse. Exclusion criteria were as follows: bone diseases, diabetes, unstable or life-threatening conditions, or requiring antibiotic therapy [29,30].

The inclusion criteria, after cause-related therapy, were as follows: full-mouth plaque score (FMPS) <25%, full-mouth bleeding score (FMBS) <25%, and high levels of compliance assessed during the cause-related therapy [31,32]. An additional inclusion criterion was that after the completion of cause-related therapy, at least one tooth with probing pocket depth (PPD) and clinical attachment level (CAL) of at least 4 mm in at least 2 measurements associated with at least 1-wall intrabony defect of at least 2 mm or a bone crater had to be present. The selected experimental periodontal area (one area per patient) had to be treated with periodontal resective surgery for therapeutic predictive reasons [8,9,33].

Another exclusion criterion was the presence of detectable plaque and bleeding on probing (BoP) in more than 25% of the experimental teeth at the end of cause-related therapy.

### 2.2. Periodontal Procedures and Clinical Measurements

The study was designed in four steps.

The first step comprised the patients’ assessment, case history, dental screening, motivation, and professional oral hygiene. A custom cause-related therapy was applied for each patient [34]. Periodontal state was evaluated and treated with cause-related therapy including oral hygiene motivation and instruction, and coronal and subgingival scaling, as needed. Emergencies, such as pain or acute dental–periodontal infections, were treated. Caries and endodontic therapy were performed, and poorly contoured restorations were corrected, as needed.

The second step was re-evaluation after the completion of cause-related therapy. The FMPS was assessed by using a plaque disclosing gel, and the percentage of total surfaces (6 aspects per tooth: mesio-buccal—MB, buccal—B, disto-buccal—DB, disto-lingual—DL, lingual—L, and mesio-lingual—ML) displaying the plaque presence was recorded. Using a periodontal probe with 1-mm marks (modified Click-Probe, Kerr Corp., Bioggio, Switzerland), we assessed BoP (6 aspects per tooth) at the considered surgical site, whereas FMBS (6 aspects per tooth) was assessed on the full mouth [35]. Before surgery, and 3 and 6 months after surgery, the subsequent clinical measurements were recorded at the experimental site: (1) PPD measured at 6 aspects per tooth (as the subsequent indices); (2) recession depth (REC); (3) CAL; (4) BoP and FMBS; (5) FMPS. Plaque detection, as well as bleeding on probing, is identified according to its presence or absence (in case of presence, 1, or absence, 0). If all requirements were met, patients signed the informed consent form and were recruited into the study. All these data were considered baseline data.

The third step was the resective surgery. The flap project was carefully planned before the surgical procedure in order to position the gingival margin at the level of the reshaped osseous crest on the basis of the dimension of gingiva, of the periodontal defect, and of the anatomy of bone crest and neighboring teeth. The fiber retention technique implies that the coronal level of the periodontal fibers is to be considered as the bottom of the intrabony defect. The position of the primary internal beveled incision was determined by the context of probing depths and the conformation of the defect and of involved teeth. The incision had to predict the position of the remodeled bone crest whether an increase of apico-coronal dimension of gingiva was or not required. The primary incision (usually a split-thickness) was apically extended. A secondary intra-sulcular incision was carried out with the blade parallel to the tooth axis. Then, the secondary flap was gently removed and the fiber system identification was performed using a periodontal probe [8,9]. The bony defect was corrected by fiber retention technique, and a positive bony architecture was obtained. Periosteal anchorage was obtained through suturing to allow for flap stability; to choose the selected position of the flap; and to reduce the coagulum between flap, periosteum, or bone surface [36]. However, immediately before the surgery, patients were randomly allocated to group A (CHX + ADS), group B (CHX), or group C (placebo).

Randomization was carried out through a computer that generated random codes, using random permuted blocks with a block size of 4 to avoid uneven splits. Allocation was concealed, by using opaque envelopes, from the surgeon until completion of the surgery. Moreover, the opening of envelopes was after completion of the common part of treatment, all clinical measurements (baseline included) were performed by a double-blind examiner (S.L.), and statistical analysis was performed by a double-blind statistician (M.L.).

In the fourth step, after surgery, the post-surgical and plaque control instructions were given to patients in relation to their respective randomly assigned group. However, it was prescribed to all patients to avoid substantial mechanical strains and masticatory efforts in relation to the treated area during the first postoperative week. No surgical dressing was used. As much as possible, patients were advised not to chew on the side of the treated area.

All patients were given specific oral hygiene instructions to gently wipe the treated area three times a day, starting the day after surgery up to the second week, with a post-surgical toothbrush (Megasoft Surgical, Curaprox, Curaden, Kriens -Switzerland). From the second week, the first toothbrush was replaced by a second, more compact, but still soft, toothbrush (Ultrasoft 5460; Curaden, Kriens-Switzerland) until the fourth week after surgery. All patients were instructed to immerse the toothbrush in chlorhexidine or placebo solution (i.e., the mouthwash or placebo, according to group assignment) and used to wipe the dento-gingival area with light vertical strokes. At week 4, after surgery, patients were re-instructed to resume normal hygiene procedures, including full interproximal cleaning and to discontinue chlorhexidine/placebo mouth rinsing.

### 2.3. Experimental Groups

Group A (ADS + CHX): Patients were instructed to rinse twice daily (morning and evening) with chlorhexidine (10 mL for 1 min, Curasept ADS^®^ 0.12%, Curasept s.p.a., Saronno, VA, Italy) for the first 4 postoperative weeks. This group could be considered as a control group because it represented the most frequent clinical use of CHX.

Group B (CHX): Patients were instructed to rinse twice daily (morning and evening) with chlorhexidine (10 mL for 1 min -PERIO-AID^®^ 0.12%; Dentaid s.r.l., Bologna, Italy) for the first 4 postoperative weeks.

Group C (P): Patients were instructed to rinse twice daily (morning and evening) with placebo (10 mL for 1 min, Sterile apyrogenic water; Eurospital s.p.a., Trieste, Italy) for the first 4 postoperative weeks. Sutures were removed at 1-week post-surgical control. Post-surgical full prophylaxis was delivered weekly during the first month after the surgical procedure, and later was delivered monthly, without using chlorhexidine.

**Figure 1:**
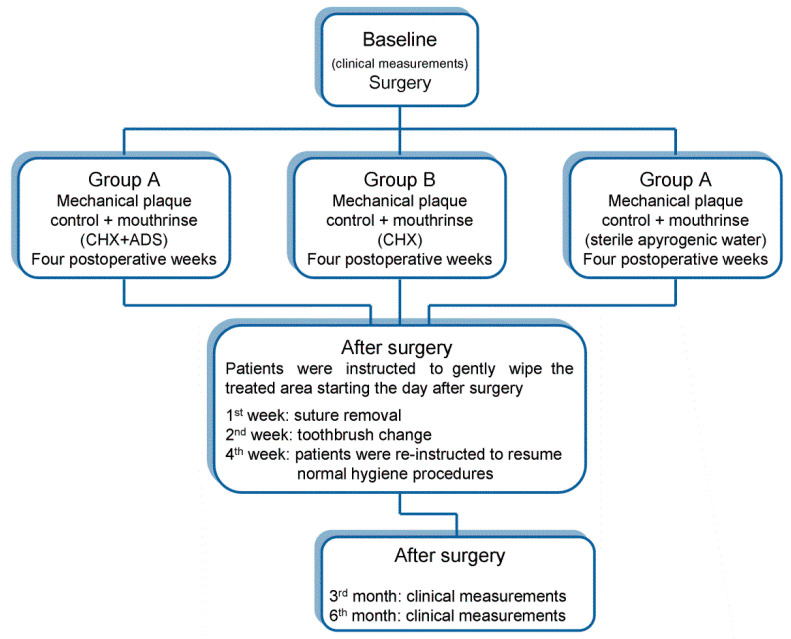
Flow chart illustrating the main points of the study.

Post-surgical controls were performed at months 3 and 6 after the surgical procedure (Figure 1). All the periodontal indices detected during the second step (baseline) were again detected at 3- and 6-month controls and compared. Moreover, differences from baseline to 3 and 6 months (differential in recordings) for FMPS; FMBS; and all the respective PPD, REC, and CAL indices were compared among the 3 groups. The study had a 6-month follow-up.

Subjects who did not meet the inclusion criteria or did not follow the protocol during the full study were dropped out.

### 2.4. Statistical Analysis

The explorative data analyses, including subgroup analyses, were applied. At the first step, the comparisons of subgroups were carried out through the multivariate analysis of covariance (MANCOVA) to verify the existence of significant differences between the examined factors and not to inflate the first type error. In fact, the involved F test in MANCOVA is sufficiently robust with respect to violations of the homoscedasticity and normality assumptions.

The second step occurred if the MANCOVA test resulted in being significant, consisting of the univariate nonparametric Kruskal–Wallis test for each dependent variable, which was necessary to reduce the inflation of the first type error again and useful to avoid the normality assumption of the used dependent variable [37,38]: FMBS, FMPS, PPD, REC, CAL.

The third step occurred if the Kruskal–Wallis test was significant and entailed the Wilcoxon–Mann–Whitney tests [38] to compare pairs of independent groups or un-matched data [37,38].

As regards the variations over time, the (non-parametric) Wilcoxon matched-pairs signed-rank test, for repeated measures of the type before–after, was used, considering the differences between the values at time = 0 (baseline) and those at 3 months, and between the values at time = 0 (baseline) and those at 6 months. Hence, the analyses proceeded as mentioned above: first, the MANCOVA tests for the differences; second, the Kruskal–Wallis tests for the univariate differences; and third, the Wilcoxon–Mann–Whitney tests.

For qualitative variables, specifically BoP, the applied tests were the chi squared test of independence, the McNemar test, and/or the equivalent binomial tests for changes over time using exact *p*-values. However, the size of groups was too small to achieve satisfactory results in the evaluation of intragroup changes.

For all measured variables, the null hypothesis (H_0_) of no difference among groups was rejected for a critical significance level of *p* < 0.05.

## 3. Results

A total of 33 patients were screened and enrolled. One was excluded immediately before surgery because an oncologic disease was immediately diagnosed. Two were excluded during the follow-up period because they did not comply with the study rules.

A total of 30 patients, 22 women and 8 men aged 34 to 70 years (mean ± SD, 53.8 ± 9.2 years), fulfilled the stepwise criteria of the study. Ten patients were randomly assigned to group A, 8 women and 2 men aged 47 to 69 years (m ± SD, 54 ± 6.7 years). Ten patients were assigned to group B, 7 women and 3 men aged 41 to 70 years (m ± SD, 56.3 ± 10.6 years), and ten patients, 7 women and 3 men aged 35-69 years (51.1 ± 10.1 years, m ± SD), were assigned to group C. Among the groups, no significant difference (*p* > 0.05) was found for age (Kruskal–Wallis test) and gender (chi-squared test).

### 3.1. Baseline

Table 1 and Table S1 (Supplementary Material) report the values of indices of groups A, B, and C at baseline and for each patient.

At baseline, all-patient (ungrouped) values (data in brackets are expressed as mean ± SD) were the FMPS and FMBS range of 10% to 23.1% (15.3 ± 3.2%) and 2% to 14.4% (9.6 ± 3.4%), respectively. The PPD, REC, and CAL-MB ranges were 1 to 8 mm (4.3 ± 1.7 mm), 0 to 3 mm (0.3 ± 0.8 mm), and 1 to 11 mm (4.6 ± 2.2 mm), respectively. The PPD, REC, and CAL-B ranges were 1–6 mm (2.8 ± 1.2 mm), 0–4 mm (0.5 ± 1.1 mm), and 1–9 (3.3 ± 1.8 mm), respectively. The PPD, REC, and CAL-DB ranges were 2 to 9 mm, (4.7 ± 1.6 mm), 0 to 4 mm (0.4 ± 0.9 mm), and 2 to 10 mm (5.1 ± 1.9 mm), respectively. The PPD, REC, and CAL-DL ranges were 2 to 9 mm (5 ± 1.7 mm), 0 to 3 mm (0.3 ± 0.8 mm), and 2 to 9 mm (5.3 ± 2.1 mm), respectively. The PPD, REC, and CAL-L ranges were 1 to 8 mm (3 ± 1.7 mm), 0 to 3 mm (0.3 ± 0.7 mm), and 1 to 11 mm (3.3 ± 2.1 mm), respectively. The PPD, REC, and CAL-ML ranges were 1 to 8 mm (4.2 ± 1.7 mm), 0 to 3 mm (0.2 ± 0.6 mm), and 1 to 11 (4.4 ± 2.1 mm), respectively. BoPs of the population, range 0 to 1, were (m ± SD) 0.3 ± 0.4 BoP-MB, 0 ± 0.2 BoP-B, 0.3 ± 0.5 BoP-DB, 0.3 ± 0.5 BoP-DL, 0 ± 0.2 BoP-L, and 0.2 ± 0.4 BoP-ML.

### 3.2. Three Months after Surgery

Table 1 and Table S2 (Supplementary Material) report the values of indices of groups A, B, and C at 3 months after surgery and for each patient.

At 3 months after surgery, all-patient (ungrouped) values (data in brackets are expressed as mean ± SD) were the FMPS and FMBS ranges were 8.3% to 18.7% (14 ± 2.7%) and 4.6% to 15.5% (9.7 ± 2.6%), respectively. The PPD, REC, and CAL-MB ranges were 1 to 3 mm (1.9 ± 0.6 mm), 0 to 4 mm (1 ± 0.9 mm), and 1 to 7 mm 2.9 ± 1.9 mm), respectively. The PPD, REC, and CAL-B ranges were 1 to 3 mm (1.3 ± 0.6 mm), 0 to 4 mm (1.2 ± 0.9 mm), and 1 to 7 mm (2.5 ± 1.3 mm), respectively. The PPD, REC, and CAL-DB ranges were 1 to 3 mm (2 ± 0.7 mm), 0 to 4 mm (1 ± 1 mm), and 1 to 7 mm (3 ± 1.2 mm), respectively. The PPD, REC, and CAL-DL ranges were 1 to 3 mm (2 ± 0.7 mm), 0 to 4 mm (0.8 ± 1.1 mm), and 2 to 7 mm (2.7 ± 1.2 mm), respectively. The PPD, REC, and CAL-L ranges were 1 to 3 mm (1.4 ± 0.6 mm), 0 to 4 mm (0.9 ± 1.1 mm), and 1 to 7 mm (2.3 ± 1.3 mm), respectively. The PPD, REC, and CAL-ML ranges were 1 to 3 mm (1.9 ± 0.7 mm), 0 to 4 mm (0.7 ± 0.9 mm), and 1 to 7 mm (2.6 ± 1.2 mm), respectively. BoPs (m ± SD) were 0.1 ± 0.2 BoP-MB, 0 ± 0 BoP-B, 0 ± 0 BoP-DB, 0.1 ± 0.3 BoP-DL, 0 ± 0.2 BoP-L, and 0.1 ± 0.2 BoP-ML.

### 3.3. Six Months after Surgery

Table 1 and Table S3 (Supplementary Material) report the values of indices of groups A, B, and C at 6 months after surgery and for each patient.

At 6 months after surgery, all-patient (ungrouped) values (data in brackets are expressed as mean ± SD) were the FMPS and FMBS ranges were 11.1% to 20% (14.4 ± 2.5%) and 1% to 18.7% (9.1 ± 3.4%), respectively. The PPD, REC, and CAL-MB ranges were 1 to 3 mm (1.6 ± 0.6 mm), 0 to 4 mm (0.9 ± 0.9 mm), and 1 to 7 mm (2.5 ± 1.1 mm), respectively. The PPD, REC, and CAL-B ranges were 1 to 2 mm (1.1 ± 0.3 mm), 0 to 4 mm (1.1 ± 1 mm), and 1 to 6 mm (2.2 ± 1.2 mm), respectively. The PPD, REC, and CAL-DB ranges were 1 to 3 mm (1.7 ± 0.7 mm), 0 to 4 mm (0.9 ± 0.9 mm), and 1 to 7 mm (2.6 ± 1.2 mm), respectively. The PPD, REC, and CAL-DL ranges were 1 to 3 mm (1.6 ± 0.7 mm), 0 to 4 mm (0.9 ± 1 mm), and 1 to 7 mm (2.4 ± 1.4 mm), respectively. The PPD, REC, and CAL-L ranges were 1 to 2 mm (1.1 ± 0.3 mm), 0 to 4 mm (0.9 ± 1 mm), and 1 to 6 mm (2 ± 1.2 mm), respectively. The PPD, REC, and CAL-ML ranges were 1 to 3 mm (1.5 ± 0.7 mm), 0 to 4 mm (0.8 ± 0.9 mm), and 1 to 7 mm (2.3 ± 1.2 mm), respectively. BoPs (m±SD) were 0.1 ± 0.2 BoP-MB, 0 ± 0.2 BoP-B, 0.1 ± 0.3 BoP-DB, 0.1 ± 0.2 BoP-DL, 0 ± 0.2 BoP-L, and 0 ± 0.2 BoP-ML.

**Figure 2:**
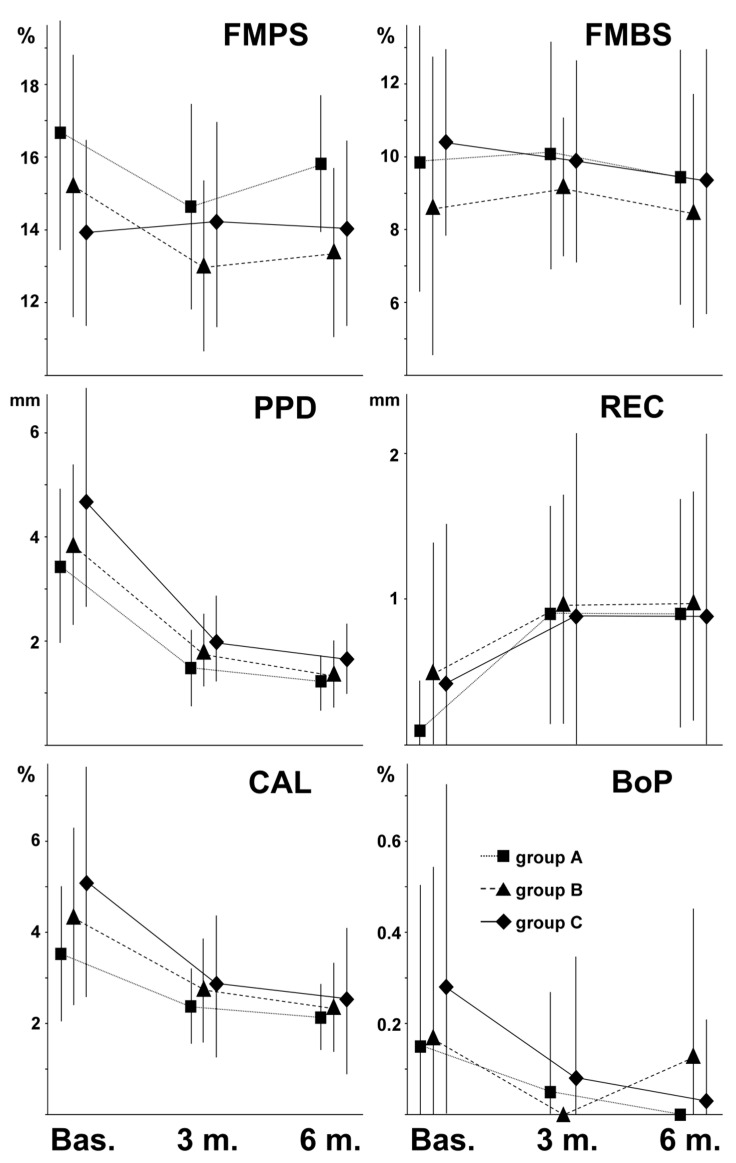
Behavior of indices (mean ± SD) of the 3 groups at baseline (Bas.) and 3 (3 m.) and 6 (6 m.) months after surgery.

Figure 2 shows the overall FMPS, FMBS, PPD, REC, CAL, and BoP behavior (mean ± SD of PPD, CAL, REC, and BoP, -MB, -B, -DB, -DL, -L and -ML measurements) of the three different groups of patients, from baseline to 6 months after surgery.

### 3.4. Baseline Statistical Comparisons

Comparing data of groups A, B, and C, we found that PPD-B comparison of group A with groups B and A with group C (m ± SD, A = 2 ± 0.7 mm, B = 2.9 ± 0.7 mm, C = 3.6 ± 1.4 mm) were both statistically significant (*p* < 0.05). CAL-B comparison of group A with groups B and A with group C (m ± SD, A = 2.1 ± 0.7 mm, B = 3.6 ± 1.3 mm, C = 4.3 ± 2.3 mm) were both statistically significant.

Comparisons of groups of the remaining periodontal indices were not significant.

### 3.5. Three-month Statistical Comparisons

Three months after surgery, inter-group comparisons were not statistically significant (*p* > 0.05) for all periodontal indices.

Considering the difference in the recordings between baseline and 3 months after surgery, we found that only CAL-B comparison between group A with C data (m ± SD at baseline, A = 2.1 ± 0.7 mm, C = 4.3 ± 2.3 mm) was statistically significant: the average CAL-B decreasing of group C was 1.6 mm, whereas no CAL-B decreasing was achieved in group A.

### 3.6. Six-Month Statistical Comparisons

The difference in PPD-MB between groups A and C and groups B and C were statistically significant. The A, B, and C averages of recorded PPD-MB were (mean ± SD) 1.4 ± 0.5 mm, 1.4 ± 0.5 mm, and 2 ± 0.5 mm, respectively. Groups A and B showed means that were statistically equal, and both differed significantly from C, as this may be argued considering the reported values.

Considering the difference in the recordings between baseline and 6 months after surgery, we found that only PPD-B and CAL-B comparison between group A (m ± SD at baseline, PPD-B = 2 ± 0.7 mm, CAL-B = 2.1 ± 0.7 mm) with group C (m ± SD at baseline, PPD-B = 3.6 ± 1.4 mm, CAL-B = 4.3 ± 2.3 mm) data were statistically significant. The average PPD-B decreasing of group C was 2.4 mm, whereas the PPD-B decreasing in group A was 1 mm. The average CAL-B decreasing of group C was 2 mm, whereas the CAL-B decreasing of group A was 0.1 mm.

### 3.7. Intragroup Comparison

From baseline to 3 and 6 months after surgery, no significant changes were observed in FMPS and FMBS of the entire patient population (Figure 2). On the contrary, particularly from baseline to 3 months after surgery, PPD and CAL were significantly decreased, and REC significantly increased (Figure 2). From baseline to 3 and 6 months after surgery, BoP significantly decreased (Figure 2), but only at -MB, -DB, and -DL measurements.

From baseline to 3 months after surgery, comparing the single groups A, B, and C, we found that the results of the entire study population were confirmed in each group, with the exception of REC and BoP. The increase of REC-ML of group A; REC-MB, -B, -DB, -DL, and -L of group B; and REC-MB, -B, -DL, and -L, of group C were not significant. The exceptions for CAL were CAL-MB, -B, and -L of group A.

Sample sizes of the groups were too low to determine significant changes. However, BoP tended to disappear, only as a trend, almost always in groups A, B, and C.

From baseline to 6 months after surgery, comparing the single groups A, B, and C, the results of the entire study population confirmed that obtained in each group, with the exception of REC, CAL, and BoP. The increase of REC-DB of group A, REC-MB, -B, -DB, -DL, -L, and -ML of groups B and C were not significant. The decrease of CAL-B and CAL-L of group A was, likewise, not significant. As in the previous case, BoP showed a tendency to disappear in all the groups.

## 4. Discussion

The in vitro studies upon the CHX action could be meaningless because oral ecosystem structures and role are extremely variable, coming under individual features (e.g., guest’s response). The bacterial cultures do not act like ecosystems. Moreover, during in vitro studies, bacterial cultures are continuously and not extemporaneously (such as during a rinsing procedure) subjected to CHX [39,40]. Some clinical studies showed too short follow-ups [41,42], and moreover, they quite often did not demonstrate that CHX rinsing was able to improve clinical results in treating septic oral inflammation [41,43]. Thus, the clinical advantage of CHX rinse associated with early use of mechanical devices for tooth cleaning is largely indeterminate. Clinical studies that indicate the real benefit of CHX use itself are lacking, not in the least because CHX rinse was at first studied to complement, not to replace, mechanical therapy [24,44,45].

Sanz et al. [14] compared post-surgical protocol using or not using CHX rinses while periodontal dressing and mechanical cleansing were performed after surgery in all cases. The use of CHX rinses was shown to be a clinically effective adjunct. However, it was to be considered that periodontal dressings could avoid mechanical trauma and reduce post-surgical pain (they quite often contains antalgic drugs) but are bulky and usually obstruct the entry to surgical sites, promoting accumulation of materials and microbiota and preventing dental brushing of the surgically treated zone. Thus, only a liquid, such as a mouth rinse, could pass under the periodontal dressing to disinfect and dilute accumulated materials. About 15 years later, Heitz et al. [27] studied two post-surgical protocols: one based on the use of only CHX mouth-rinses in the control group, and the other, in addition to CHX rinsing, patients applied CHX gel locally using a very soft surgical toothbrush from days 3 to 14, and a soft toothbrush from days 14 to 28, twice daily. The test protocol based on CHX rinses plus mechanical cleansing seemed to be recommended. Therefore, the mechanical plaque control performed correctly after surgery seems to guarantee clinical favorable outcomes. However, both the protocols used CHX.

ADS is quite often added to CHX to avoid teeth discoloration, thus resulting in being more tolerated than CHX rinse alone. However, ADS addition could decrease CHX effectiveness [46]

In this study, periodontal resective surgery was chosen, considering that is a well-known method to obtain predictive periodontal pocket resolution. Moreover, this kind of surgery reduces operative variables, having a limited number of methodologies with the use of graft not usually being considered [8,9]. The cause-related therapy is influenced by a large number of variables depending on local, systemic, and individual distinguishing features. Therefore, custom protocols were applied to achieve a good clinical outcome [34]

Periodontal surgery of late years has become minimally invasive, reducing flap extension and volume [7,8,9,47]. The use of minimally invasive criteria together with suture techniques and advanced materials has given rise to more stable flaps [7,47]. Moreover, the increased awareness of oral hygiene significance, of home dental care, and of oral hygiene instruction [48,49,50] has led to the development of patients’ compliance and home hygiene aids, as well as increasingly refined hygienic techniques in recent years. Professional hygiene procedures, implemented during post-surgical supportive therapy, have been refined. Periodontal dressing is less used, and dental care at home procedures are attainable to every compliant patient. Thus, current periodontal surgical and post-surgical hygienic techniques are able to support tooth-brushing involved in the surgical site without the risk to damage suture and tissues.

Our study took into account three groups of patients who were similar to each other in terms of gender and age, as well as in terms of similar systemic and periodontal conditions. All the enrolled patients had to be compliant. However, this criterion is now a required standard in periodontal surgery [48], and thus enrolled patients represent an ordinary population of periodontal patients who underwent periodontal surgery also from this point of view.

At baseline, PPD-B and CAL-B showed a clinically better condition, statistically significant in group A in comparison with groups B and C. However, 3 months and 6 months after surgery, no statistically significant differences were observed as PPD-B and CAL-B among the groups. Considering the difference in the recordings between baseline and 3 months after surgery, we found that CAL-B comparison between group A and group C was statistically significant: the average CAL-B decreasing of group C was 1.6 mm, whereas no CAL-B decreasing was achieved in group A. CAL-B showed an average value of 1.2 mm as CAL gain in group C, with a corresponding average PPD-B and REC-B of 1.6 mm and 1.1 mm, respectively. Moreover, considering the difference in the recordings, only PPD-B and CAL-B comparison between group A and group C was statistically significant 6 months after surgery. The average PPD-B decreasing in group C was 2.4 mm, whereas it was 1 mm in group A. In group C, CAL-B gain was 1.6 mm, whereas it was −0.8 mm in group A. The average PPD-B was 1.0 and 1.2 mm in groups A and C, respectively. A greater clinical recovery was observed in group C, and it allowed for the achievement of physiological clinical conditions such as PPD and to obtain a noticeable CAL-gain. Moreover, the differences in PPD-MB between groups A and C, and groups B and C, were statistically significant only 6 months after surgery. However, the greater PPD-MB average was only 2 mm (2 ± 0.5 mm). It should be considered that healthy crevicular sulcus depth is ≤ 3 mm [51].

Considering the intragroup periodontal indices behavior, from baseline to 3 and 6 months after surgery, no significant changes were observed in FMPS and FMBS of the full patient population. On the contrary, PPD and CAL significantly decreased, and REC significantly increased, in particular from baseline to 3 months after surgery. BoP also decreased, at least as a trend. The enrolled patients were compliant during the entire study follow-up, and periodontal indices showed the usual healing pattern after the periodontal surgery.

Also considering the CAL-gain in comparison between the groups, we found that the CAL-gain was nearly always observed 6 months after surgery, with the only exception of CAL-B-gain in group A. The average PPD-B and REC-B in group A baseline were 2 and 0.1 mm, respectively; nonetheless, it is to be considered that this site did not require a particular CAL-gain as a rule. Moreover, the selected cases always underwent resective surgery, and thus a limited degree of gingival recession was expected.

In terms of CAL-gain, we was naturally considered the CAL-difference in the recordings between baseline and 3 months or 6 months, subtracting the REC-difference between 3 and 6 months after surgery, respectively. Thus, CAL-gain really corresponded to the periodontal attachment gain.

The worst periodontal conditions, in terms of numeric analysis of PPD, were recorded in the -MB, -ML, -DB, and -DL sites; on the contrary, the greater conceivable efficiency of mechanical hygiene (implemented without the aid of dental floss and interdental brushes) was considered in the -B and -L aspects where toothbrushes could work more effectively. However, excellent clinical results were obtained for the entire experimental population without statistical differences among groups A, B, and C, which showed the same concurrent clinical significance to both index analyses (between baseline and 3 months and baseline and 6 months) and in the analysis of the difference in the recordings.

## 5. Conclusions

This study showed that periodontal resective surgery performed on compliant patients followed by only mechanical plaque control produces excellent clinical results that are comparable to that obtained by combining mechanical and chemical control (with or without ADS system) of oral ecosystems. No clinically significant differences were demonstrated among the three groups of the considered patients 3 months after surgery and at the end of the follow-up period. The brushing alone had as good an outcome after surgery, and thus it was possible to obtain the same clinical outcome in the postoperative period without the side effects of CHX.

## Figures and Tables

**Table 1:** Indices of groups A, B, and C (mean ± SD).

Cheking Times	Groups	FMPS	FMBS	PPD	REC	CAL	BoP
Baseline	A	16.67 ± 3.01	9.85 ± 3.62	3.43 ± 1.49	0.10 ± 0.30	3.53 ± 1.48	0.15 ± 0.36
	B	15.24 ± 3.59	8.63 ± 4.06	3.85 ± 1.57	0.50 ± 0.89	4.35 ± 2.02	0.17 ± 0.38
	C	13.93 ± 2.63	10.40 ± 2.61	4.67 ± 2.09	0.42 ± 1.08	5.08 ± 2.53	0.28 ± 0.45
3 months	A	14.64 ± 2.86	10.08 ± 3.16	1.48 ± 0.70	0.90 ± 0.73	2.38 ± 0.74	0.05 ± 0.22
	B	13.03 ± 2.37	9.20 ± 1.88	1.80 ± 0.66	0.97 ± 0.80	2.77 ± 1.09	0.00 ± 0.00
	C	14.22 ± 2.81	9.89 ± 2.80	1.98 ± 0.70	0.88 ± 1.30	2.87 ± 1.66	0.08 ± 0.28
6 months	A	15.82 ± 1.87	9.44 ± 3.52	1.23 ± 0.50	0.90 ± 0.75	2.13 ± 0.72	0.00 ± 0.00
	B	13.42 ± 2.42	8.49 ± 3.18	1.38 ± 0.56	0.98 ± 0.75	2.37 ± 1.01	0.13 ± 0.34
	C	14.03 ± 2.58	9.36 ± 3.69	1.65 ± 0.66	0.88 ± 1.30	2.53 ± 1.67	0.03 ± 0.18

FMPS and FMBS are expressed as a percentage; PPD, REC, and CAL are expressed as millimeters; BoP: presence = 1, absence = 0. PPD, REC, CAL, and BoP respective -MB, -B, -DB, -DL, -L, and -ML measurements are reported on the 6 consecutive lines (from top to bottom).

## Data Availability

Data is contained within the article or supplementary material.

## Supplementary Materials

The following are available online at https://www.mdpi.com/article/10.3390/ma14112933/s1, Table S1: Groups A, B and C indices at baseline, Table S2: Groups A, B and C indices at 3 months after surgery, Table S3: Groups A, B and C indices at 6 months after surgery
