# Supplementary material for: Influence of Tooth-Brushing on Early Healing after Access Flap Surgery: A Randomized Controlled Preliminary Study
Source: Materials (Basel). 2021 May 29;14(11):2933. doi: 10.3390/ma14112933 (PMC8198661; doi:10.3390/ma14112933)
Supplement: Supplementary file 1 [file materials-14-02933-s001.zip › materials-1214222-supplementary materials-publish.pdf]

materials

MDPI

Supplementary materials

Influence of Tooth-Brushing on Early Healing after Access Flap Surgery: A Randomized Controlled Preliminary Study

Carlo Bertoldi [1,*], Luigi Generali [1,*], Pierpaolo Cortellini [2], Michele Lalla [3], Sofia Luppi [4], Aldo Tomasi [1], Davide Zaffe [5,*], Roberta Salvatori [6] and Stefania Bergamini [1]

1 Department of Surgery, Medicine, Dentistry and Morphological Sciences with Transplant Surgery, Oncology and Regenerative Medicine Relevance, University of Modena and Reggio Emilia, 41124 Modena, Italy; aldo.tomasi@unimore.it; (A.T.); stefania.bergamini@unimore.it (S.B.)
2 The European Research Group on Periodontology (ERGOPerio), 3855 Brienz - Bern, Switzerland; sandro@cortellini.org
3 Department of Economics Marco Biagi, University of Modena and Reggio Emilia, 41121 Modena, Italy; michele.lalla@unimore.it
4 Independent Researcher, 41124 Modena, Italy; sofialuppi@outlook.it
5 Department of Biomedical, Metabolic and Neural Sciences, University of Modena and Reggio Emilia, 41125 Modena, Italy
6 Biomaterials Laboratory, Department of Medical and Surgical Sciences of Children and Adults, University of Modena and Reggio Emilia, 41124 Modena, Italy; roberta.salvatori@unimore.it
* Correspondence: carlo.bertoldi@unimore.it (C.B.); luigi.generali@unimore.it (L.G.); davide.zaffe@unimore.it

Table S1. Groups A, B and C indices at baseline.

A Group	CL	DR	IA	AP	IF	RG	GI	SC	NM	GC
FMPS	19.2	15	13.4	17.3	15	14.9	16.6	23.1	13.5	18.7
FMBS	8.7	14	11.6	9.6	12.5	5.5	3.2	7.4	13.5	12.5
PPD	4	1	2	5	4	3	4	3	6	4
	2	1	2	3	2	2	2	1	3	2
	4	4	4	6	4	5	4	6	3	4
	4	4	5	7	4	5	4	7	3	4
	2	1	3	4	3	2	2	1	3	2
	4	1	2	5	4	3	3	4	5	5
REC	0	0	0	0	0	0	0	0	0	0
	0	0	0	0	0	0	1	0	0	0
	1	0	0	0	0	0	0	0	0	0
	1	0	0	0	0	0	0	0	0	0
	0	0	0	0	0	1	1	0	0	0
	0	0	0	0	0	1	0	0	0	0
CAL	4	1	2	5	4	3	4	3	6	4
	2	1	2	3	2	2	3	1	3	2
	5	4	4	6	4	5	4	6	3	4
	5	4	5	7	4	5	4	7	3	4
	2	1	3	4	3	3	3	1	3	2
	4	1	2	5	4	4	3	4	5	5
BoP	0	0	1	1	0	0	0	0	0	0
	0	0	0	0	0	0	0	0	0	0
	0	0	1	0	1	0	0	1	0	0
	0	0	0	1	1	0	0	0	0	0
	0	0	0	0	0	0	0	0	0	0
	0	0	0	0	0	1	0	0	0	1
**B group**	**LS**	**RG**	**AC**	**GF**	**LM**	**LC**	**TC**	**MZ**	**AD**	**PI**
FMPS	10	13.6	13	17.2	18.5	15.3	21	10	17.1	16.7
FMBS	3	11.5	9	10.4	13	5.8	2	14.4	7.9	9.3

PPD	2	4	5	2	8	4	4	6	5	4
	3	3	4	2	2	3	4	3	3	2
	4	4	3	4	9	4	5	2	6	4
	4	4	3	5	8	5	3	2	6	4
	2	3	3	3	3	3	4	3	3	2
	2	4	5	2	6	4	2	6	5	4
REC	0	0	0	0	1	0	0	0	3	1
	0	0	0	1	1	0	0	0	4	1
	0	1	0	0	1	0	0	0	3	1
	0	0	0	0	3	0	0	0	1	1
	0	0	0	0	2	0	0	0	1	1
	0	0	0	0	1	0	0	0	1	1
CAL	2	4	5	2	9	4	4	6	8	5
	3	3	4	3	3	3	4	3	7	3
	4	5	3	4	10	4	5	2	9	5
	4	4	3	5	11	5	3	2	7	5
	2	3	3	3	5	3	4	3	4	3
	2	4	5	2	7	4	2	6	6	5
BoP	0	0	0	0	1	0	0	1	0	0
	0	0	0	0	0	0	0	0	0	0
	0	0	0	0	1	0	0	0	0	0
	0	1	0	0	1	1	0	1	0	0
	0	0	0	0	0	0	0	1	0	0
	0	0	0	1	0	0	0	1	0	0
**C group**	**AC**	**AB**	**IM**	**MS**	**TF**	**FI**	**ES**	**CP**	**LZ**	**AC**
FMPS	14.1	10.8	15	10.4	16.6	17.8	12	17.1	13	12.5
FMBS	10.9	10	7	5.2	10	13.4	12	13.5	11.1	10.9
PPD	5	4	4	5	5	7	1	8	4	5
	4	2	2	3	4	6	5	5	2	3
	9	5	4	4	4	6	8	3	4	5
	9	5	4	4	5	6	7	7	3	8
	5	2	2	3	1	5	1	8	2	8
	5	3	4	4	4	8	2	8	4	7
REC	0	0	0	0	1	0	0	3	0	0
	0	0	0	3	0	0	0	4	0	0
	0	0	0	1	0	0	0	4	0	0
	0	0	0	0	0	0	0	3	0	0
	0	0	0	0	0	0	0	3	0	0
	0	0	0	0	0	0	0	3	0	0
CAL	5	4	4	5	6	7	1	11	4	5
	4	2	2	6	4	6	5	9	2	3
	9	5	4	5	4	6	8	7	4	5
	9	5	4	4	5	6	7	10	3	8
	5	2	2	3	1	5	1	11	2	8
	5	3	4	4	4	8	2	11	4	7
BoP	0	0	1	0	1	1	0	1	0	0
	0	0	0	0	0	0	1	0	0	0
	1	1	1	0	1	1	0	0	0	0
	1	0	1	0	1	1	0	0	0	0
	0	0	0	0	0	0	0	0	0	0
	0	0	0	0	1	1	0	1	0	0

FMPS and FMBS are expressed as a %; PPD, REC and CAL are expressed as mm; BoP: presence = 1, absence =0. PPD, REC, CAL and BoP respective -MB, -B, -DB, -DL, -L and -ML measurements are reported on the 6 consecutive lines (from top to bottom)

Table S2. Groups A, B and C indices at 3 months after surgery.

**A Group**	**CL**	**DR**	**IA**	**AP**	**IF**	**RG**	**GI**	**SC**	**NM**	**GC**
GFMPS	16.3	16.6	16.3	16	17.5	8.3	14.8	13.9	11.1	15.6
FMBS	8.7	11.1	12	10	15	6.7	4.6	9.3	9.3	14.1
PPD	1	1	1	3	1	2	2	2	2	1
	1	1	1	1	1	1	1	1	1	1
	1	1	1	3	1	2	2	3	2	1
	1	1	2	2	2	2	2	3	2	1
	1	1	1	1	1	1	1	1	2	1
	1	1	1	3	1	2	1	3	3	1
REC	1	1	1	0	2	2	1	0	1	2
	1	1	1	0	2	2	2	0	0	2
	1	1	1	0	2	2	1	0	0	2
	1	1	2	0	1	1	0	0	0	1
	1	1	2	0	1	2	1	0	0	1
	1	1	1	0	1	1	0	0	0	1
CAL	2	2	2	3	3	4	3	2	3	3
	2	2	2	1	3	3	3	1	1	3
	2	2	2	3	3	4	3	3	2	3
	2	2	4	2	3	3	2	3	2	2
	2	2	3	1	2	3	2	1	2	2
	2	2	2	3	2	3	1	3	3	2
BoP	0	0	0	1	0	0	0	0	0	0
	0	0	0	0	0	0	0	0	0	0
	0	0	0	0	0	0	0	0	0	0
	0	0	0	1	1	0	0	0	0	0
	0	0	0	0	0	0	0	0	0	0
	0	0	0	0	0	0	0	0	0	0
**B group**	**LS**	**RG**	**AC**	**GF**	**LM**	**LC**	**TC**	**MZ**	**AD**	**PI**
FMPS	11.5	13	13.3	15.3	16.3	11.1	14.3	9.6	15.7	10.2
FMBS	5.7	10.9	8.3	9.6	11.3	9.2	7.1	11.5	10.12	8.3
PPD	2	2	3	2	3	2	1	3	2	2
	1	1	2	1	2	1	1	2	2	1
	2	2	3	2	3	2	1	1	2	2
	1	2	2	2	3	2	1	2	2	1
		1	2	2	2	1	1	1	2	1
	2	2	2	2	3	2	1	3	2	1
REC	1	1	1	0	2	0	1	2	1	1
	1	1	1	1	2	1	1	1	2	2
	2	2	0	0	2	0	1	0	2	1
	2	1	0	0	3	0	1	0	1	1
	1	1	0	0	3	0	1	0	1	2
	0	1	1	0	2	0	1	0	1	1
CAL	3	3	4	2	5	2	2	5	3	3
	2	2	3	2	4	2	2	3	4	3
	4	4	3	2	5	2	2	1	4	3
	3	3	2	2	6	2	2	2	3	2
	2	2	2	2	5	1	2	1	3	3
	2	3	3	2	5	2	2	3	3	2

BoP	0	0	0	0	0	0	0	0	0	0
	0	0	0	0	0	0	0	0	0	0
	0	0	0	0	0	0	0	0	0	0
	0	0	0	0	0	0	0	0	0	0
	0	0	0	0	0	0	0	0	0	0
	0	0	0	0	0	0	0	0	0	0
**C group**	**AC**	**AB**	**IM**	**MS**	**TF**	**FI**	**ES**	**CP**	**LZ**	**AC**
FMPS	12.5	11.4	16.6	10	13	12.5	14.2	18.7	16.1	17.2
FMBS	9.2	7.8	7.5	6.2	9.3	8.9	9.5	15.5	12.5	12.5
PPD	2	2	2	2	2	2	1	3	2	2
	2	1	1	1	2	1	3	3	1	1
	3	2	2	2	2	2	3	3	3	1
	3	2	2	2	3	3	2	3	2	1
	2	1	2	2	2	1	1	3	1	1
	2	2	2	2	2	3	2	3	1	2
REC	1	1	1	1	0	0	0	4	0	0
	1	1	0	3	0	1	1	4	0	0
	1	1	1	2	0	0	1	4	0	0
	0	0	0	0	0	0	0	4	0	3
	1	1	0	0	0	0	0	4	0	3
	1	0	0	0	0	1	0	4	0	2
CAL	3	3	3	3	2	2	1	7	2	2
	3	2	1	4	2	2	4	7	1	1
	4	3	3	4	2	2	4	7	3	1
	3	2	2	2	3	3	2	7	2	4
	3	2	2	2	2	1	1	7	1	4
	3	2	2	2	2	4	2	7	1	4
BoP	0	0	0	0	0	0	0	1	0	0
	0	0	0	0	0	0	0	0	0	0
	0	0	0	0	0	0	0	0	0	0
	0	1	0	0	0	0	0	0	0	0
	0	0	1	0	0	0	0	0	0	0
	0	0	0	0	0	0	0	1	0	1

FMPS and FMBS are expressed as a %; PPD, REC and CAL are expressed as mm; BoP: presence = 1, absence = 0. PPD, REC, CAL and BoP respective -MB, -B, -DB, -DL, -L and -ML measurements are reported on the 6 consecutive lines (from top to bottom).

Table S3. Groups A, B and C indices at 6 months after surgery.

**A Group**	**CL**	**DR**	**IA**	**AP**	**IF**	**RG**	**GI**	**SC**	**NM**	**GC**
FMPS	18.2	17.8	14.1	16.6	15	13	13.9	14.8	17.6	17.2
FMBS	3.4	7.8	10.9	12.5	15	7.4	6.4	7.4	11.1	12.5
PPD	1	1	1	2	1	1	2	2	2	1
	1	1	1	1	1	1	1	1	1	1
	1	1	1	3	1	1	1	2	2	1
	1	1	1	2	2	1	1	2	1	1
	1	1	1	1	1	1	1	1	1	1
	1	1	1	3	1	1	1	2	1	1
REC	1	1	0	0	2	2	1	0	1	2
	1	1	0	0	2	2	2	0	0	2
	1	1	0	0	2	2	1	0	0	2
	1	1	1	0	2	2	1	0	0	1
	1	1	1	0	2	1	1	0	0	1
	1	1	1	0	2	1	1	0	0	1

CAL	2	2	1	2	3	3	3	2	3	3
	2	2	1	1	3	3	3	1	1	3
	2	2	1	3	3	3	2	2	2	3
	2	2	2	2	4	3	2	2	1	2
	2	2	2	1	3	2	2	1	1	2
	2	2	2	3	3	2	2	2	1	2
BoP	0	0	0	0	0	0	0	0	0	0
	0	0	0	0	0	0	0	0	0	0
	0	0	0	0	0	0	0	0	0	0
	0	0	0	0	0	0	0	0	0	0
	0	0	0	0	0	0	0	0	0	0
	0	0	0	0	0	0	0	0	0	0
**B group**	**LS**	**RG**	**AC**	**GF**	**LM**	**LC**	**TC**	**MZ**	**AD**	**PI**
FMPS	16.3	12	12.5	11.5	18	13.5	15.7	11.1	12.5	11.1
FMBS	9.3	9.8	9.2	7.7	13	7.8	1	6.5	10.4	10.2
PPD	2	1	2	1	1	1	1	2	2	1
	1	1	1	1	1	1	1	1	2	1
	2	2	2	1	2	1	1	1	2	1
	2	1	2	1	3	1	2	1	2	1
	1	1	1	1	2	1	1	1	2	1
	2	1	2	1	3	1	1	1	2	1
REC	0	1	1	0	2	1	1	1	0	1
	2	2	1	1	2	1	0	0	2	2
	1	2	0	0	2	1	1	0	1	1
	2	1	0	0	2	1	2	0	1	1
	2	1	0	1	2	1	2	0	0	2
	1	1	1	0	2	1	1	0	0	1
CAL	2	2	3	1	3	2	2	3	2	2
	3	3	2	2	3	2	1	1	4	3
	3	4	2	1	4	2	2	1	3	2
	4	2	2	1	5	2	4	1	3	2
	3	2	1	2	4	2	3	1	2	3
	3	2	3	1	5	2	2	1	2	2
BoP	0	0	1	0	0	0	0	0	0	0
	0	0	1	0	0	0	0	0	0	0
	0	0	1	0	0	0	0	0	1	0
	0	1	1	0	0	0	0	0	0	0
	0	0	1	0	0	0	0	0	0	0
	0	0	1	0	0	0	0	0	0	0
**C group**	**AC**	**AB**	**IM**	**MS**	**TF**	**FI**	**ES**	**CP**	**LZ**	**AC**
FMPS	11.7	11.9	15.2	12.5	14.8	12	14.4	20	12.2	15.6
FMBS	8.3	7.1	8	8.3	6.5	5.6	9.6	18.7	10.6	10.9
PPD	2	2	2	2	2	2	1	3	2	2
	1	1	1	1	1	1	2	2	1	1
	3	2	2	2	2	2	2	3	2	1
	3	2	2	2	1	2	1	3	1	1
	1	1	1	1	1	1	1	2	1	1
	2	2	2	2	1	2	1	3	1	1

REC	1	1	1	1	0	0	0	4	0	0
	1	1	0	3	0	1	1	4	0	0
	1	1	1	2	0	0	1	4	0	0
	0	0	0	0	0	0	0	4	0	3
	1	1	0	0	0	0	0	4	0	3
	1	0	0	0	0	1	0	4	0	2
CAL	3	3	3	3	2	2	1	7	2	2
	2	2	1	4	1	2	3	6	1	1
	4	3	3	4	2	2	3	7	2	1
	3	2	2	2	1	2	1	7	1	4
	2	2	1	1	1	1	1	6	1	4
	3	2	2	2	1	3	1	7	1	3
BoP	0	0	0	0	0	0	0	1	0	0
	0	0	0	0	0	0	0	0	0	0
	0	0	0	0	0	0	0	1	0	0
	0	0	0	0	0	0	0	0	0	0
	0	0	0	0	0	0	0	0	0	0
	0	0	0	0	0	0	0	0	0	0

FMPS and FMBS are expressed as a %; PPD, REC and CAL are expressed as mm; BoP: presence = 1, absence = 0. PPD, REC, CAL and BoP respective -MB, -B, -DB, -DL, -L and -ML measurements are reported on the 6 consecutive lines (from top to bottom).
